# Vitamin D and tuberculosis: a multicenter study in children

**DOI:** 10.1186/s12879-014-0652-7

**Published:** 2014-12-11

**Authors:** Elisabetta Venturini, Ludovica Facchini, Nuria Martinez-Alier, Vas Novelli, Luisa Galli, Maurizio de Martino, Elena Chiappini

**Affiliations:** Department of Health Sciences, University of Florence, Anna Meyer Children’s University Hospital, viale Pieraccini 24, Florence, I-50139 Italy; Evelina London Children’s Hospital, Guy’s and St Thomas’ NHS Foundation Trust, London, UK; Department of Infectious Diseases, Great Ormond Street Hospital for Children NHS Trust, Great Ormond Street, London, WC1N 3JH UK

**Keywords:** Vitamin D, Tuberculosis, Children

## Abstract

**Background:**

The aim of this study is to evaluate vitamin D levels in children with latent and active TB compared to healthy controls of the same age and ethnical background.

**Methods:**

A multicenter observational study has been conducted in three tertiary care paediatric centres: Anna Meyer Children's University Hospital, Florence, Italy; Evelina London Children's Hospital, London, United Kingdom and Great Ormond Street Hospital, London, United Kingdom. Vitamin D was considered deficient if the serum level was <25 nmol/L, insufficient between 25 and 50 nmol/L and sufficient for a level >50 nmol/L.

**Results:**

The study population included 996 children screened for TB, which have been tested for vitamin D. Forty-four children (4.4%) had active TB, 138 (13.9%) latent TB and 814 (81.7%) were controls. Our study confirmed a high prevalence of hypovitaminosis D in the study population. A multivariate analysis confirmed an increased risk of hypovitaminosis D in children with latent and active TB compared to controls [(P = 0.018; RR = 1.61; 95% CI: 1.086-2.388), (P < 0.0001; RR = 4.587; 95% CI:1.190-9.608)].

**Conclusions:**

Hypovitaminosis D was significantly associated with TB infection in our study. Further studies are needed to evaluate a possible role of vitamin D in the treatment and prevention of tuberculosis in children.

**Electronic supplementary material:**

The online version of this article (doi:10.1186/s12879-014-0652-7) contains supplementary material, which is available to authorized users.

## Background

In the pre-antibiotic era, cod liver oil and sunlight exposure were used to treat tuberculosis (TB) [1],[2]. More recently, increasing evidences from *in vitro* studies suggest that vitamin D enhances antimycobacterial immunity [3]. Several authors reported hypovitaminosis D in TB patients [4]–[6], and serum level of vitamin D [25-hydroxycholecalciferol) was found to be lower in TB patients than in healthy controls [7]–[14]. A recent study conducted among adult TB contacts found that 94% of recruits were vitamin D insufficient and that a single, oral 2.5 mg dose of vitamin D significantly enhanced their anti-mycobacterial immunity in vitro [7]. Factors such as low socioeconomic status, poor nutrition, traditional/cultural traits, and little exposure to sunlight may contribute to vitamin D deficiency [15].

Mechanisms through which vitamin D modulates the immune system in the response to *Mycobacterium tuberculosis* infection are not completely understood, two possible mechanisms have emerged as the most likely. Vitamin D appears to reduce the viability of *M. tuberculosis* by enhancing the fusion of the phagosome and lysosome in infected macrophages [16]. In addition, vitamin D may enhance the production of LL-37, an antimicrobial peptide of the cathelicidin family [16]–[19]. Antimicrobial peptides, such as defensin and cathelicidin, are involved as a first line of defences in the prevention of infections, including tuberculosis. The presence of vitamin D in neutrophils and macrophages up-regulates in a dose-dependent manner the hCAP-18 gene that codes for LL-37, which suggests that vitamin D induction of LL-37 may play a role in host defences against TB infection [3],[16].

Vitamin D exerts its actions through vitamin D receptor (VDR), a nuclear hormone receptor. Polymorphisms in the VDR gene, which may influence VDR activity and subsequent downstream vitamin D mediated effects, were therefore studied as potential candidates of risk markers for various clinical outcomes [20],[21].

Clinical trials [8],[10],[22]–[24] have been conducted to test whether vitamin D therapy improves TB outcomes, suggesting that vitamin D may be beneficial as an adjunctive treatment to the traditional therapy in patients with TB; however, additional trials in children need to be conducted to clarify its role [25].

## Methods

A multicenter study was conducted in three tertiary care paediatric centres: Anna Meyer Children's University Hospital, Florence, Italy; Evelina London Children's Hospital, London, United Kingdom and Great Ormond Street Hospital, London, United Kingdom. The data regarding the period of time between July 2008 and January 2013 were collected retrospectively, whereas those between February and September 2013 prospectively. Written informed consent for participation in the study was obtained from children's parent or guardian. The aim of the study was to evaluate vitamin D levels in children with latent and active tuberculosis, compared to healthy controls of the same age and ethnical background. Secondary objective was to evaluate potential differences between groups of patients (considering age, race, enrolling centre, vitamin D supplementation, latent or active tuberculosis).

### Study design

Children (aged <18 years) investigated for TB infection between July 2008 and September 2013, for whom vitamin D was tested during the first visit, were eligible. The reasons for referral to the paediatric infectious diseases centres were clinical suspicious or confirmed TB disease, history of TB contact or immigrated/adopted child from a TB endemic country within the previous 2 years. Children with congenital or acquired immunodeficiency were excluded.

For each child the following data were entered into the study database: name, age, gender, ethnic group, familial and personal history including risk factors for TB infection and for vitamin D deficiency, *bacillus* Camette-Guerin (BCG) vaccination and physical examination. Data about tests performed were also included, in particular vitamin D level (25-hydroxycholecalciferol, 25-OHD), tuberculin skin test (TST), interferon-γ release assay (IGRA), and if available calcium, phosphate, chest x-ray, chest computerised tomography, gastric aspirate or sputum (microscopy, culture and polymerase chain reaction for *M. tuberculosis*) and anti-tubercular treatment. All results were recorded in the study database following the international standards for the protection of privacy and personal information.

### Ethical issues

This study was approved by the Ethical Committees of the hospitals involved: Anna Meyer Children's University Hospital, Florence, Italy; Evelina London Children's Hospital, London, United Kingdom and Great Ormond Street Hospital, London, United Kingdom.

### Case definitions

#### TB disease

Active TB diagnosis was assigned to any child with *Mycobacterium tuberculosis* cultured or detected by microscopy or molecular methods from sputum, gastric aspirate or other biologic samples. Active TB diagnosis was also assigned to any child with clinical and radiological evidence of TB disease, and with either a history of exposure to an infectious case or a positive TST. In the absence of a recognized gold standard, latent tuberculosis diagnosis was assigned to any child with a positive TST and/or IGRA and no clinical, bacteriological or radiographic evidence of active TB [26]. In the absence of those criteria children were included in the control group.

#### Hypovitaminosis D

Vitamin D level was evaluated testing for 25-OHD, which is considered its best indicator [27]. Following the European Society for Paediatric Gastroenterology, Hepatology and Nutrition (ESPGHAN) definition, 25-OHD was considered deficient in case of a serum vitamin D level less than 25 nmol/L, insufficient between 25 and 50 nmol/L and sufficient for level above 50 nmol/L [28].

### Statistical analysis

Continuous measurements analyzed were: age (years), weight (kilograms), height (centimetres), 25-OHD serum level (nmol/L), calcium and phosphate levels (mmol/L). Median and interquartile range (IQR) were calculated for those variables in the study groups. Categorical data were compared using the Chi-squared test (or Fisher's exact test, when expected cell sizes were smaller than five). The Wilcoxon-Mann–Whitney test was used for continuous measurements to test relationships in unpaired analysis, when assumed that the dependent variable was a not normally distributed interval variable. Moreover, the risk factors for vitamin D deficiency were evaluated using univariate and multivariate logistic regression. The variables included in the analysis were: TB infection status (active TB, latent TB, and control), gender, age, ethnicity at risk for vitamin D deficiency, immigration, seasonality (categorized ad autumn-winter and spring-summer). For each factor the relative risk (RR) and 95% confident intervals (95% CI) were evaluated. Statistical analysis was performed using the statistical software SPSS for Windows, version 12.0. P <0.05 was considered statistically significant.

## Results

### Population

Nine hundred and ninety-six children were included in this study: 15 (1.5%) from Great Ormond Street Hospital, London, UK; 63 (6.4%) from Evelina London Children’s Hospital, London, UK; and 918 (92.1%) from Anna Meyer Children's University Hospital, Florence, Italy. The study population characteristics are summarized in Table 1.

**Table 1:** **Study population characteristics**

	Controls n = 814	Latent TB n = 138	Active TB n = 44	Total n = 996	P
**Enrolling centre, n (%):**					<0.0001
*- Evelina London Children’s*	21 (2.6%)	25 (18.1%)	17 (38.6%)	63 (6.4%)	
*Hospital, London, UK*					
*- Great Ormond Street*	0 (0%)	0 (0%)	15 (34.1%)	15 (1.5%)	
*Hospital, London, UK*					
*- Anna Meyer Children's University Hospital, Florence, Italy*	793 (97.4%)	113 (81.9%)	12 (27.3 %)	918 (92.1%)	
**Age in years, median (IQR)**	5.5 (3.1-8.1)	6.9 (4.3-10.8)	4.7 (3.0-11.6)	5.8 (3.1-8.5)	<0.0001
**Gender, n (%):**					<0.0001
*- Male*	496 (60.9%)	85 (61.6%)	15 (34.1%)	596 (59.4%)	
*- Female*	310 (38.1 %)	52 (37.7%)	29 (65.9 %)	391 (39.6%)	
*- Not known*	8 (1%)	1 (0.7%)	0 (0%)	9 (0.9%)	
**Country of origin** * **, n (%):**					<0.0001
*- Asia*	146 (17.9%)	19 (13.8%)	4 (9.1%)	169 (17%)	
*- Latin America*	183 (22.5%)	24 (17.4%)	4 (9.1%)	211 (21.2%)	
*- Eastern Europe*§	269 (33%)	57 (41.3%)	5 (11.4%)	331 (33.2%)	
*- Western Europe*	36 (4.4%)	7 (5.1%)	3 (6.8 %)	46 (4.6%)	
*- Nord Africa*	16 (2%)	1 (0.7%)	2 (4.5%)	19 (1.9%)	
*- Sub-Saharan Africa*	151 (18.6%)	29 (21%)	25 (56.8 %)	205 (20.6%)	
*- Other*	8 (1%)	0 (0%)	0 (0%)	8 (0.8%)	
*- Not known*	5 (0.6%)	1 (0.7%)	1 (2.3%)	7 (0.7%)	
**Ethnic group, n (%):**					<0.0001
***-*** *African*	168 (20.7%)	30 (21.7%)	27 (61.4%)	225 (22.6%)	
*- Asiatic*	146 (17.9%)	19 (13.8%)	4 (9.1%)	169 (17%)	
*- Caucasian*	306 (37.6%)	64 (46.4%)	8 (18.2%)	378 (37.9%)	
*- Hispanic*	183 (22.5%)	24 (17.4%)	4 (9.1%)	211 (21.2%)	
*- Other*	6 (0.7%)	0 (0%)	0 (0%)	6 (0.6%)	
*- Not known*	5 (0.6%)	1 (0.7%)	1 (2.2%)	7 (0.7%)	
**BCG, n (%):**					0.109
*- Yes*	460 (56.5%)	89 (64.5%)	20 (45.5%)	569 (57.1%)	
*- No*	244 (30.0%)	40 (29.0%)	19 (43.2%)	303 (30.4%)	
*- Not known*	110 (13.5%)	9 (6.5%)	5 (11.4%)	124 (12.4%)	
**Risk factors for vitamin D**					0.932
**deficiency†, n (%):**					
*- Yes*	470 (57.7%)	74 (53.6%)	32 (72.7%)	576 (57.8%)	
*- No*	344 (42.3%)	64 (46.4%)	12 (27.3%)	420 (42.2%)	
**Risk factors for TB**					<0.0001
**infection, n (%):**					
**-** *Tb contact*	92 (11.4%)	31 (22.5%)	27 (21.2%)	150 (15.1%)	
**-** *Travel to/immigrated from*	695 (85.3%)	104 (75.4%)	10 (22.8%)	809 (80.1%)	
*TB endemic country*					
*- Not known*	27 (3.3%)	3 (2.1%)	7 (15.9%)	37 (3.7%)	
**IGRA, n (%):**					<0.0001
*- Positive*	0 (0.0%)	45 (32.6%)	28 (63.6%)	73 (7.3%)	
*- Negative*	792 (97.3%)	71 (51.4%)	7 (15.9%)	870 (87.3%)	
*- Not known*	22 (2.7%)	22 (15.9%)	9 (20.5%)	53 (5.3%)	
**TST, n (%):**					<0.0001
*- Positive*	0 (0.0%)	120 (87%)	32 (72.7%)	152 (15.3%)	
*- Negative*	777 (95.5%)	10 (7.2%)	2 (4.5%)	789 (79.2%)	
*- Not known*	37 (4.5%)	8 (5.8%)	10 (22.7%)	55 (55.2%)	

*Whether the child’s family is originally from a TB endemic country, this has been indicated as country of origin, independently from the country of birth.

§Albania, Belarus, Bulgaria, Kosovo, Macedonia, Moldavia, Poland, Czech Republic, Romania, Russia, Serbia, Slovakia, Slovenia, Ukraine, Hungary.

**†**African, Asiatic and Hispanic ethnic group.

### TB status

Forty-four (4.4%) children met the criteria for active TB. The most common condition was pulmonary TB (n = 24; 54.5%). Extra-pulmonary TB was in order of frequency: lymphadenitis (n = 5; 11.4%), pleural-peritoneal (n = 4; 9.1%), skeletal (n = 4; 9.1%), ocular (n = 1; 2.3%), meningeal (n = 1; 2.3%). Miliary TB was found in 5 patients (11.4%). The median length of the treatment was 7 months (range 6–12). Quadruple therapy (isoniazid, rifampicin, ethambutol, pyrazinamide) was used in 36 children, in 4 of those in association with steroids. In other 8 cases different anti-TB treatment associations have been used, mostly because of resistant mycobacteria.

One hundred and thirty-eight (13.9%) children met the criteria for latent TB. Double therapy (isoniazid and rifampicin) for 3 months was the commonest combination used (91.3%, n = 126). The controls enrolled in the study were 814 (81.7%).

### Vitamin D, calcium and phosphate levels in the study groups

**Figure 1:**
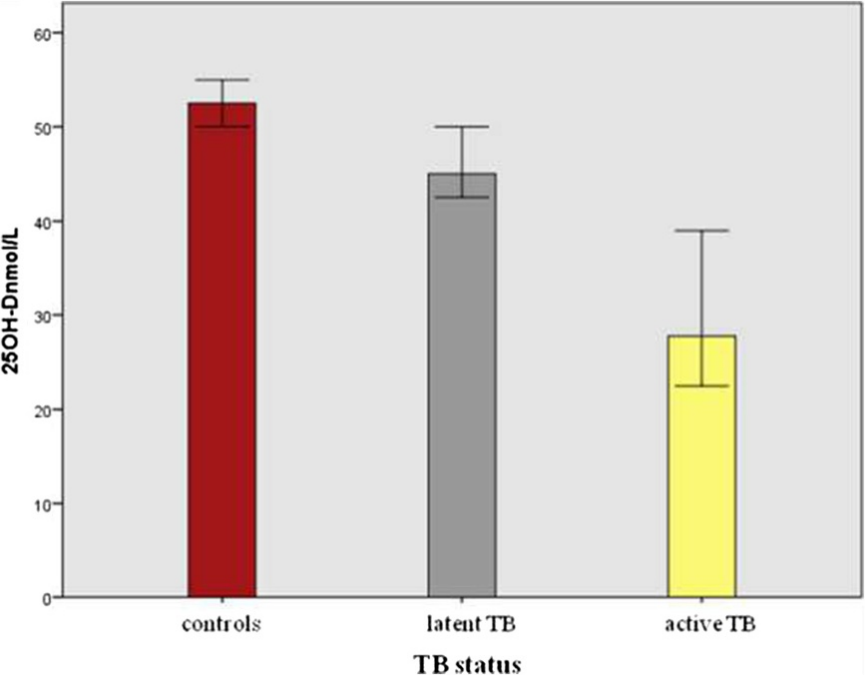
**Vitamin D levels (median and IQR) in the different study groups.** About half (467; 46.9%) of the children tested, independently from the TB status, resulted to have an insufficient or deficient vitamin D level. Hypovitaminosis D was found respectively in 354 (43.5%) of controls, 80 (58%) latent TB and 33 (75%) active TB.

Vitamin D levels in the different study groups are reported in Table 2 and illustrated in Figure 1. About half (467; 46.9%) of the children tested, independently from the TB status, resulted to have an insufficient or deficient vitamin D level. Hypovitaminosis D was found respectively in 354 (43.5%) of controls, 80 (58%) latent TB and 33 (75%) active TB. The statistical analysis with the Mann–Whitney U test showed that vitamin D level was significantly lower in case of latent and active TB compared to controls (p < 0.0001), with median level respectively of 45 nmol/L (IQR: 30–62.5), 27.8 nmol/L (IQR: 19–50) and 52.5 nmol/L (IQR: 31.5-67.5).

**Table 2:** **Vitamin D, calcium and phosphate levels in the different study groups**

	Controls n = 814	Latent TB n = 138	Active TB n = 44	Total n = 996	P
**Vitamin D level, n (%):**					0.0001
***-*** *Deficient (<25 nmol/L)*	113 (13.9%)	28 (20.3%)	18 (40.9%)	159 (16%)	
*- Insufficient (25–50 noml/L)*	241 (29.6%)	52 (37.7%)	15 (34.1%)	308 (30.9%)	
*- Sufficient (>50 nmol/L)*	460 (56.5%)	58 (42%)	11 (25%)	529 (53.1%)	
**Vitamin D level (nmol/L), median (IQR)**	52.5 (31.5-67.5)	45 (30–62.5)	27.8 (19–50)	35 (24–54.5)	0.0001
**Calcium (mmol/L), median (IQR)**	2.38 (2.3-2.45)	2.38 (2.3-2.42)	2.35 (2.25-2.45)	2.4 (2.26-2.46)	0.709
**Phosphate (mmol/L), median (IQR)**	1.62 (1.49-1.71)	1.75 (1.32-1.78)	1.42 (1.23-1.6)	1.5 (1.28-1.61)	0.002

Vitamin D levels were significantly lower in latent TB compared to controls (p = 0.002), in active TB compared to controls (p < 0.0001) and in active TB compared to latent TB (p = 0.001).

**Figure 2:**
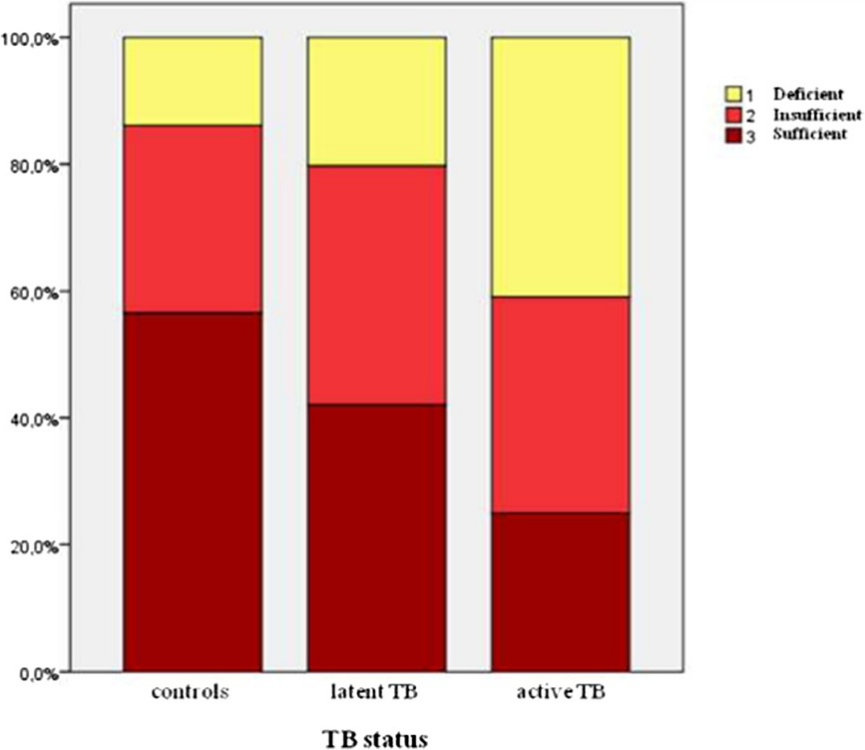
**Vitamin D status defined by ESPGHAN according to TB status.** A deficient vitamin D level was found in higher percentage in the active TB group (n = 18; 40.9%) compared to latent TB (n = 28; 20.3%) and controls (13.9%) (P < 0.0001). An insufficient level of vitamin D was more frequently found in the latent TB group (n = 113; 37.7%, P < 0.0001).

Moreover, a deficient vitamin D level was found in higher percentage in the active TB group (n = 18; 40.9%) compared to latent TB (n = 28; 20.3%) and controls (13.9%) (P < 0.0001). An insufficient level of vitamin D was more frequently found in the latent TB group (n = 113; 37.7%, P < 0.0001) (Figure 2).

All the variable analysed were statistically significant also according to the US Endocrinology Society vitamin D status classification (P < 0.0001) (29).

Calcium and phosphate levels (median and IQR) in the different study groups are reported in Table 2.

In the study population 159 (16%) children were vitamin D deficient, 308 (30.9%) insufficient and 529 (53.1%) sufficient.

The correlation between vitamin D status and other factors (age, gender, seasonality, calcium and phosphate levels) has been reported in Table 3.

**Table 3:** **Characteristics of the population according to vitamin D status**

	Deficient n = 159	Insufficient n = 308	Sufficient n = 529	Total n = 996	P
**TB status, n (%):**					<0.0001
*- Controls*	113 (71.1%)	241 (78.2%)	460 (87%)	814 (81.7%)	
*- Latent TB*	28 (17.6%)	52 (16.9%)	58 (11%)	138 (13.9%)	
*- Active TB*	18 (11.3%)	15 (4.9%)	11 (2%)	44 (4.4%)	
**Enrolling centre, n (%):**					<0.0001
*- Evelina London Children’s*	26 (16.4%)	23 (7.5%)	14 (2.6%)	63 (6.4%)	
*Hospital, London, UK*					
*- Great Ormond Street*	8 (5%)	5 (1.6%)	2 (0.4%)	15 (1.5%)	
*Hospital, London, UK*					
*- Anna Meyer Children's University Hospital, Florence, Italy*	125 (78.6%)	280 (90.9%)	513 (97%)	918 (92.1%)	
**Age, median (IQR)**	6.5 (4.2-10.3)	6.6 (4–9.7)	4.8 (2.5-7.6)	5.8 (3.1-8.5)	<0.0001
**Gender, n (%):**					0.106
*- Male*	85 (53.5%)	182 (59.1%)	329 (62.2%)	596 (59.8%)	
*- Female*	74 (46.5%)	122 (39.6%)	195 (36.9%)	391 (39.3%)	
*- Not known*	0 (0%)	4 (1.3%)	5 (0.9%)	9 (0.9%)	
Country of origin*, n (%):					<0.0001
*- Asia*	27 (17%)	44 (14.3%)	98 (18.5%)	169 (17%)	
*- Latin America*	25 (15.7%)	74 (24%)	112 (21.2%)	211 (21.2%)	
*- Eastern Europe* §	48 (30.2%)	95 (30.8%)	188 (35.5%)	331 (33.2%)	
*- Western Europe*	4 (2.5%)	18 (5.8%)	24 (4.5%)	46 (4.6%)	
*- Nord Africa*	7 (4.4%)	8 (2.6%)	4 (0.8%)	19 (1.9%)	
*- Sub-Saharan Africa*	42 (26.4%)	65 (21.1%)	98 (18.5%)	205 (20.6%)	
*- Other*	1 (0.6%)	3 (1%)	4 (0.8%)	8 (0.8%)	
*- Not known*	5 (3.1%)	1 (0.3%)	1 (0.2%)	7 (0.7%)	
**Ethnic group, n (%):**					<0.0001
***-*** *African*	50 (30.8%)	74 (24%)	101 (19.1%)	225 (22.6%)	
*- Asiatic*	24 (15.1%)	43 (14%)	102 (19.3%)	169 (17%)	
*- Caucasian*	52 (32.7%)	115 (37.3%)	211 (39.9%)	378 (37.9%)	
*- Hispanic*	27 (17%)	73 (23.7%)	111 (21%)	211 (21.2%)	
*- Other*	1 (1.2%)	2 (0.7%)	3 (0.5%)	6 (0.6%)	
*- Not known*	5 (3.2%)	1 (0.3%)	1 (0.2%)	7 (0.7%)	
**Risk factors for vitamin D**					0.932
**deficiency†, n (%):**					
*- Yes*	93 (58.5%)	180 (58.4%)	303 (57.3%)	576 (57.8%)	
*- No*	66 (41.5%)	128 (41.6%)	226 (42.7%)	420 (42.2%)	
**Season when blood test was**					<0.0001
**done, n (%):**					
*- Spring-Summer*	58 (36.5%)	145 (47.1)	339 (64.1%)	542 (54.4%)	
*- Winter-Autumn*	101 (63.5%)	163 (52.9)	190 (35.9%)	454 (45.6%)	
**Vitamin D level (nmol/L), median (IQR)**	20 (15.5-22.5)	37.5 (32.5-42.5)	72.5 (57.5-87.5)	35 (24–54.5)	
**Calcium (mmol/L), median (IQR)**	2.35 (2.28-2.42)	2.37 (2.3-2.43)	2.38 (2.33-2.48)	2.4 (2.26-2.46)	0.030
**Phosphate (mmol/L), median (IQR)**	1.5 (1.3-1.65)	1.59 (1.41-1.68)	1.65 (1.49-1.81)	1.5 (1.28-1.61)	0.001

*Whether the child’s family is originally from a TB endemic country, this has been indicated as country of origin, independently from the country of birth.

§Albania, Belarus, Bulgaria, Kosovo, Macedonia, Moldavia, Poland, Czech Republic, Romania, Russia, Serbia, Slovakia, Slovenia, Ukraine, Hungary.

**†**African, Asiatic and Hispanic ethnic group.

**Figure 3:**
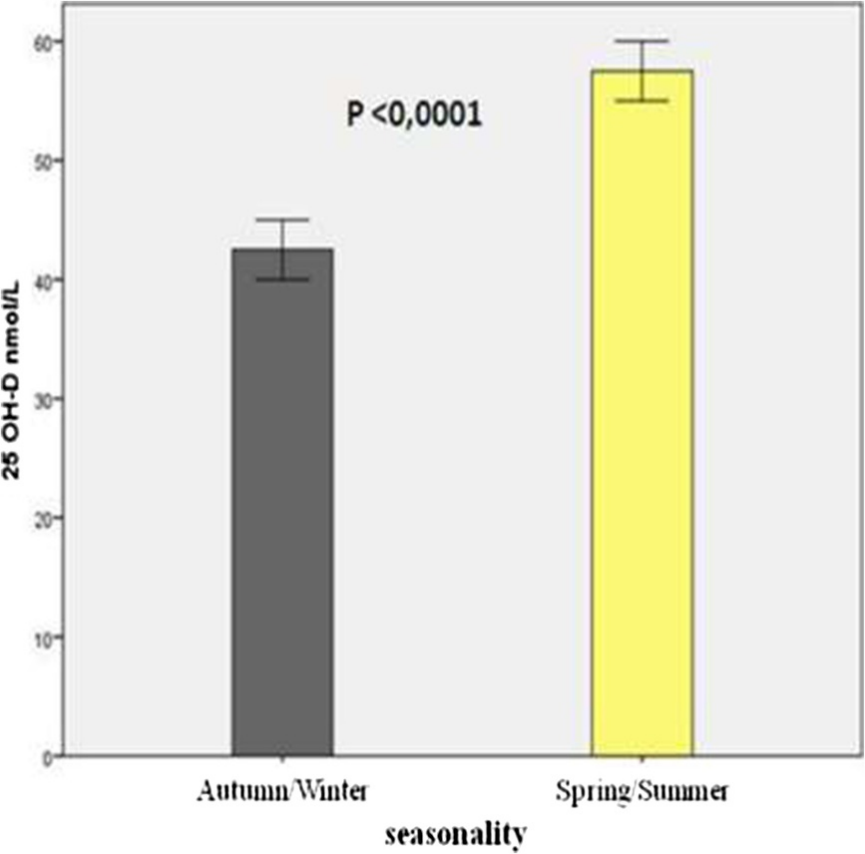
**Vitamin D level according to seasonality.** Vitamin D level was significantly lower if tested during Autumn-Winter compared to Spring-Summer (P < 0.0001).

Vitamin D level was significantly lower if tested during Autumn-Winter compared to Spring-Summer (P < 0.0001) (Figure 3).

### Multivariate analysis

A multivariate logistic regression analysis was used to exclude possible confounding factors. This analysis confirmed the risk of vitamin D deficiency to be statistically correlated with TB infection (RR = 1.61; 95% CI:1.086-2.388; P = 0.018;), and higher in active TB compared to latent TB and controls, ( RR = 4.587; 95% CI: 1.190-9.608; P < 0.0001). In the multivariate analysis the results were adjusted for the different centres.

There was no significant correlation between hypovitaminosis D and gender (P = 0.113; RR = 0.799; 95% CI: 0.605-1.055), ethnicity at risk of hypovitaminosis D (P = 0.688; RR = 1.610; 95% CI: 0.715-1.747) and immigration (P = 0.428; RR = 1.316; 95% CI: 0.668-2.590). On the contrary, a correlation between hypovitaminosis D and age (P < 0.0001; RR = 1.147; 95% CI: 1.105-1.190) and between vitamin D deficiency and seasonality (P < 0.0001; RR = 2.208; 95% CI: 2.132-3.698) was found.

### Vitamin D supplementation

In case of hypovitaminosis D, different vitamin D supplementation protocols were used in the three centres, according with the most recent international guidelines [28]–[31]. At Anna Meyer Children's University Hospital, Florence, Italy, 1000 IU of colecalciferol were administered daily for 8 weeks. At Evelina London Children’s Hospital, London, UK, 3,000, 6,000, 10,000 or 20,000 IU of colecalciferol were used daily for 6 weeks depending on the severity of hypovitaminosis D. At Great Ormond Street Hospital, London, UK 50,000 IU of ergocalciferol were administered daily for 3 to 6 days.

Only one-third (133; 28.5%) of the patients with a low level of vitamin D received vitamin D supplementation. Vitamin D supplementation was prescribed in 71 controls, 36 latent TB and 26 active TB, respectively 20%, 45% and 79% of children with hypovitaminosis D in the 3 different groups.

## Discussion

The high prevalence of hypovitaminosis D in children [32],[33] is confirmed in our study, based on a large data set, reaching 47% of children tested, independently from their TB status.

The correlation between vitamin D deficiency and TB disease was previously demonstrated in adults [4]–[6],[8]–[14], although some authors do not agree with this hypothesis [34]–[40]. No paediatric study with a matched control group is available in literature, and our study’s aim is to fill this gap. Three studies on a possible correlation between vitamin D deficiency and TB in the paediatric population were previously published [41]–[43], including overall 442 children. In the first study on refugee children, a vitamin D level was significantly lower in 92 children with TB infection compared to 236 healthy controls [41]. The main limitations of this retrospective study were the omission of routine IGRA testing in the definition of TB status and the missing data about BCG vaccination [41]. Hypovitaminosis D prevalence in latent and active TB was 86% in a retrospective study on 64 children [42]. The principal limitation of this study was the lack of an age and ethnically matched control group without a history of TB infection or disease [42]. Finally, in a randomised study, done on a small number of children (n = 24), vitamin D supplementation (colecalciferol 1000 UI daily for 8 weeks) was added to TB treatment, leading to clinical and radiological improvement compared to the standard treatment alone [43].

The correlation between low vitamin D and TB disease is confirmed in our study on a large paediatric population including a matched control group. Hypovitaminosis D affected up to 75% children with active TB (P < 0.0001). Moreover, the active TB group exhibited the lowest level of vitamin D. Hypovitaminosis D was four times more likely in children with active TB compared to healthy controls. This correlation was confirmed using a multivariate logistic regression analysis, in order to exclude possible confounding factors, and the results were adjusted considering also the different enrolling centre.

About two-third of the study population ethnicity was at high risk for vitamin D deficiency (African, Asiatic and Hispanic). It is well known that the major risk factors in these populations are dark skin, reduced exposure to sunlight due to long clothing and vegetarian diet [32]. The ethnical background at risk of hypovitaminosis D were homogeneously distributed in the 3 study groups. Interestingly, in our study hypovitaminosis D occurred also in the Caucasian group, accounting for about one-third of the cases. Vitamin D was deficient in 32.7% of Caucasian children and insufficient in 37.3%. Moreover, about 80% of children tested in the UK had hypovitaminosis D (respectively 43.6% was vitamin D deficient and 35.9% insufficient) compared to 44.1% of children tested in Italy (respectively 13.6% was vitamin D deficient and 30.5% insufficient). This difference could be mainly explained by lower sun exposure in UK with respect to Italy and probably also by different diet within the two countries. However, the high percentage of hypovitaminosis in Italy despite good sun exposure, affecting about half of the children tested, should increase the awareness of this problem also in countries known to be at low risk for vitamin D deficiency.

Hypovitaminosis D was statistically more frequent if tested during autumn and winter compared to spring and summer. This seasonality has been well described and is mainly related to sun exposure [6],[12],[41],[44].

Surprisingly, only one-third of the patients with hypovitaminosis D received vitamin D supplementation, with different protocols depending on the prescribing centre. The protocols used in the three centres were in agreement with the most recent international guidelines [28]–[31]. This data should alert the physicians about the need of vitamin D supplementation in children with hypovitaminosis, to prevent rickets and its complications. However no clinical sign of rickets was reported in our study population. The choice of vitamin D regimen in the paediatric population should take count of compliance, especially in young children and in TB patients, which already receive a significant amount of drugs. The need of clear guidelines for children with hypovitaminosis D in this setting should be addressed, in order to unify the management of vitamin D supplementation in this group of patients.

The major limitations of our study are: the lack of homogeneity between the study groups, as the majority of controls were enrolled in the Italian centre. To minimize the influence of this factor on our results a multivariate analysis was performed, including the enrolling centre as a categorical variable. Moreover data regarding parathyroid hormone, alkaline phosphatase were lacking. In our study the hypovitaminosis D definition used was in keeping with ESPGHAN guidelines [28], although we were aware about different definitions. To avoid this bias we performed the analysis considering also vitamin D status classification by the US Endocrine Society [29], and the results didn’t change substantially.

## Conclusions

Our large population study confirms an increasing incidence of hypovitaminosis D in Europe, within native and immigrated children, and the role played by vitamin D status in TB disease.

In our study hypovitaminosis D was significantly associated with TB infection. Further studies are needed to evaluate a possible role of vitamin D in the treatment and prevention of tuberculosis in children, especially novel randomized controlled trials to compare TB treatment outcomes in children receiving vitamin D supplementation in addition to the standard therapy.

## Authors’ original submitted files for images

Below are the links to the authors’ original submitted files for images. Authors’ original file for figure 1 Authors’ original file for figure 2 Authors’ original file for figure 3

## Abbreviations

25-OHD: 25-Hydroxycholecalciferol
BCG: Bacillus Calmette-Guerin
CI: Confidence intervals
ESPGHAN: European Society for Paediatric Gastroenterology, Hepatology and Nutrition
IGRA: Interferon-γ release assay
IQR: Interquartile range
RR: Relative risk
TB: Tuberculosis
TST: Tuberculin skin test
VDR: Vitamin D receptor
