# Supplementary figures and images for: Vitamin D and tuberculosis: a multicenter study in children
Source: BMC Infect Dis. 2014 Dec 11;14:652. doi: 10.1186/s12879-014-0652-7 (PMC4272523; doi:10.1186/s12879-014-0652-7)

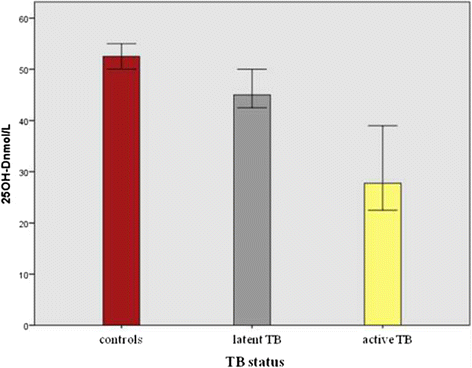

Supplement: Supplementary file 1 — Authors’ original file for figure 1 [file 12879_2014_652_MOESM1_ESM.gif]

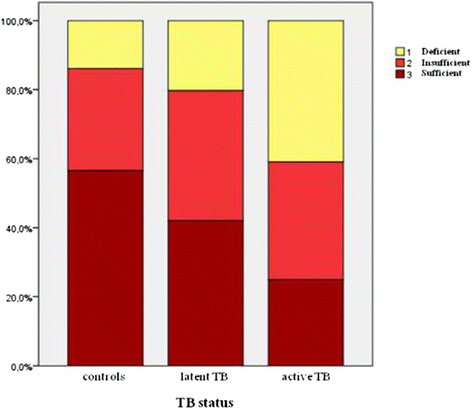

Supplement: Supplementary file 2 — Authors’ original file for figure 2 [file 12879_2014_652_MOESM2_ESM.gif]

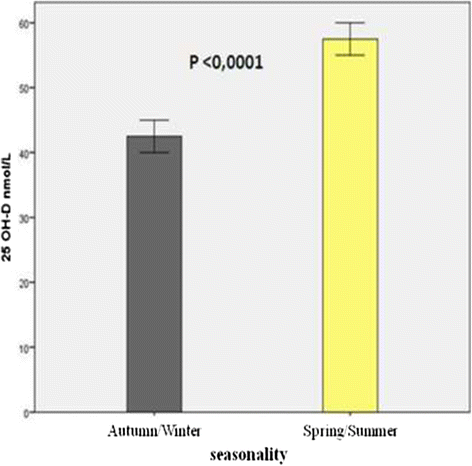

Supplement: Supplementary file 3 — Authors’ original file for figure 3 [file 12879_2014_652_MOESM3_ESM.gif]
